# Emergency surgery over 111 years: are we still at a crossroads or ready for emergency surgery 2.0?

**DOI:** 10.1186/s13049-015-0189-9

**Published:** 2015-12-21

**Authors:** Kjetil Søreide

**Affiliations:** Department of Gastrointestinal Surgery, Stavanger University Hospital, P.O. Box 8100, N-4068 Stavanger, Norway; Department of Clinical Medicine, University of Bergen, Bergen, Norway

“*Every important hospital should have on its resident staff of surgeons at least one who is well and able to deal with any emergency that may arise*”. [1]

William S. Halsted (1852–1922)

With 2015 at a close, it is now a solid 111 years that have passed since Halstead voiced his opinion in his 1904 address [1]. The statement still rings a very logical bell to everyone who either works in a hospital or ever was a patient. However, the action to ensure this has not been as logical and straightforward with each generation of surgeons having their own challenges to face. As I have addressed the topic before in this Journal [2, 3], and elsewhere [4, 5], it is with some reluctance (“do I need to address this topic again?”) yet still with a feel of need (“where are we now?”) that I take to the pen.

First, and regrettably, emergency cases are still a neglected field in most countries, despite acknowledged high mortality numbers and increasing documentation of care issues [6–10]. The several explanations to the discrepancies between need and provision are many and include political, societal issues as well as those related to education and training. The changes seen in medicine, with a predominant “omics” focus and personalized medicine may not fit immediately with unpredictable, out-of-hours presentations for which resources are fewer and outcomes are worse. Let alone the vanishing training in general surgery, for the better good for highly complex surgery procedures that benefit from isolated focus, yet separates the “generalism” of the past from the narrow focus of the present. Together with restricted working-hours, it becomes apparent that the exposures in training are influenced. Notably, most of these changes are for the better good of patients, but the groups that lack organized interest groups, a patients advocacy organization and defined or named funding institutes will have to live off the crumbles left after supra-specialized fields have had their say. Alas, the decibel democracy may jeopardize the right to care for some patients, and this is enhanced when the proper specialist, specialty or department does not even have a name and address from where to voice their views. Thus, “emergency surgery” needs to be defined, given a name and a place in every organization structure lest it be neglected and served as a stepchild compared to its more ‘prominent’ siblings. Exactly how, what and where is less important (e.g. tailored to the institutions need and service), as long as it will have a name and place on the institution map.

Second, as follows from the above, fight over resources and operating rooms needs a better organized approach to the patient presenting with an emergency condition in need of care [11]. Highly efficient systems are possible and well-documented [12], as are the effects of creating specialized units from several different parts of the world [9, 13–16]. There is ample evidence to show that ring-fencing of elective surgery from emergency care, creates cost-efficient pathways for both patient groups, with reduced disturbances, less cancellations and speedier and more efficacious management. So why not do it?

Third, it appears that the development of a “new” specialty is what is done to address the need for focused attention to emergency surgical conditions, as general surgery as we know it is vanishing. In Norway, it appears that a new specialty structure and remodelling of training will dismiss “general surgery” as a defined specialty overall, and incorporate trauma and acute surgery into the current specialty of gastrointestinal surgery. While this may be a logical step from the current training situation and a reasonable logic in terms of disease burden and exposure to conditions, it will require a restructured training approach in many aspects, including time, focused areas of training, procedures and hospital volume and population coverage. However, the exact form and content is yet to be chiselled and currently only a rough template appears in view. Notably, The European Union of Medical Specialists (UEMS) is now launching opportunity for Fellowship examinations in Emergency surgery starting April 2016 (prior to the ECTES 2016 conference in Vienna; see http://www.uemssurg.org/divisions/emergency-surgery/ebsq/how-to-apply). Successful candidates will achieve the title of Fellow of European Board of Surgery – Emergency surgery (FEBS/EmSurg). This is a step forward in standardizing curriculum and criteria across Europe.

Fourth, change in demography has already occurred, but will continue to heavily influence disease burden, workload and outcome in the years to come. The geriatric and multimorbid patient is known to every health care system, and these patients will increasingly present with emergency conditions and trauma. Diseases of age, including chronic organ dysfunction that worsens with acute on-set disease, will put a considerable burden on health systems and demand new ways of managing the patients [17, 18]. The age-specific incidences of acute disease and trauma – including fractures [19], perforated hollow viscus abdominal organs [20] and ruptured abdominal aortic aneurysms [21] – suggest that the numbers will increase even further in the near future.

Fifth, patients will have a greater say in the future, with greater need for openness and patient or next-of-kin inclusion in decision-making and the options available. This behoves an even greater experience-base, knowledge and skillset on behalf of the surgeon in charge to find the best solutions and tailor treatment to what is in the patient’s best interest. Ethics will clearly be more prominently visible and a tangible topic throughout patients’ care and we should learn to set a high standard.

Finally, we should focus on research in emergency surgery and trauma, both the quality and the quantity, as this is the fundament upon which we lay our decisions for optimal patient care. It is troubling to see the void of research into classical surgical themes such as appendicitis (e.g. what causes the disease in the first place?) [22] or perforated peptic ulcers (so many patients, but so few trials!) [23], although clinical progress is being made and dogmas challenged (e.g. antibiotics as primary treatment for uncomplicated appendicitis) [24]. The need for international collaboration is obvious and has many facets and opportunities beyond randomized trials that should be explored [25]. One such project is the current ongoing GlobalSurg [26] that has recruited over 10,000 patients undergoing emergency abdominal surgery for which results will soon be published. Also, this study is followed in its second form, the GlobalSurg2 focusing on surgical site infections worldwide.

Thus, there are new and exciting opportunities ahead for those interested to get involved in a highly challenging, diverse, yet rewarding area of medicine and surgery. Indeed, the whole concept of emergency surgery and trauma may be on of the most clinically interesting and academically rewarding areas in surgery for both the near and distant future. The bid is out, no go bite at it. Let us make Emergency general surgery 2.0 the service under which we all would want to receive care ourselves, be it as a severely injured or as an elderly with an acute surgical condition. Let us make Emergency surgery 2.0 work!
